# Accurate Initial State Estimation in a Monocular Visual–Inertial SLAM System

**DOI:** 10.3390/s18020506

**Published:** 2018-02-08

**Authors:** Xufu Mu, Jing Chen, Zixiang Zhou, Zhen Leng, Lei Fan

**Affiliations:** School of Optics and Photonics, Beijing Institute of Technology, Beijing 100081, China; muxufu@163.com (X.M.); zhouzixiang@bit.edu.cn (Z.Z.); lengzhen@bit.edu.cn (Z.L.); 2120170527@bit.edu.cn (L.F.)

**Keywords:** visual–inertial SLAM, initial state estimation, termination criterion

## Abstract

The fusion of monocular visual and inertial cues has become popular in robotics, unmanned vehicles and augmented reality fields. Recent results have shown that optimization-based fusion strategies outperform filtering strategies. Robust state estimation is the core capability for optimization-based visual–inertial Simultaneous Localization and Mapping (SLAM) systems. As a result of the nonlinearity of visual–inertial systems, the performance heavily relies on the accuracy of initial values (visual scale, gravity, velocity and Inertial Measurement Unit (IMU) biases). Therefore, this paper aims to propose a more accurate initial state estimation method. On the basis of the known gravity magnitude, we propose an approach to refine the estimated gravity vector by optimizing the two-dimensional (2D) error state on its tangent space, then estimate the accelerometer bias separately, which is difficult to be distinguished under small rotation. Additionally, we propose an automatic termination criterion to determine when the initialization is successful. Once the initial state estimation converges, the initial estimated values are used to launch the nonlinear tightly coupled visual–inertial SLAM system. We have tested our approaches with the public EuRoC dataset. Experimental results show that the proposed methods can achieve good initial state estimation, the gravity refinement approach is able to efficiently speed up the convergence process of the estimated gravity vector, and the termination criterion performs well.

## 1. Introduction

In recent years, visual SLAM has reached a mature stage, and there exist a number of robust real-time systems or solutions [1,2,3]. Vision-based approaches can estimate simultaneously the six-degrees-of-freedom (6-DOF) state of sensors and reconstruct a three-dimensional (3D) map of the environment. The concept of using one camera has become popular since the emergence of MonoSLAM [4], which is based on the extended Kalman filter (EKF) framework and is able to achieve real-time localization and mapping indoors in room-sized domains. After this, there have been many scholarly works on monocular visual SLAM, including PTAM [1], SVO [5], and ORB-SLAM2 [6]. PTAM [1] is the first optimization-based solution to split tracking and mapping into separate tasks processed in two parallel threads. However, similarly to many earlier works, it can only work in small scenes and easily suffers from tracking loss. ORB-SLAM2 [6] takes advantages of PTAM and further improves it. Up to now, ORB-SLAM2 has been the most reliable and complete solution for monocular visual SLAM. Although monocular visual SLAM has made great achievements in localization and mapping, it is a partially observable problem, in which sensors do not offer the depth of landmarks. To address these problems, a common and effective solution is to fuse IMU and visual measurements using filter- or optimization-based frameworks.

Many promising monocular visual–inertial SLAM systems have been proposed in recent years, such as MSCKF [7], visual–inertial ORB-SLAM2 [8] and the monocular VINS applied for micro aerial vehicles (MAVs) [9]. A tightly coupled fusion strategy jointly optimizes sensor states of the IMU and camera, which takes into account correlations between the internal states of both sensors. Along with the promotion of computing power and the use of sliding windows, nonlinear optimization and tightly coupled methods [10,11,12] have attracted great interest among researchers in recent years because of their good trade-off between accuracy and computational efficiency. Compared with filtering [13,14,15] tightly fusion frameworks, the optimization-based approaches provide better accuracy for the same computational task [16]. However, the performance of state-of-the-art nonlinear monocular visual–inertial systems [8,9,10,17,18,19] heavily relies on the accuracy of initial estimated states, which include visual scale, gravity, IMU biases and velocity. A poor initial state estimation will decrease the convergence speed or even lead to completely incorrect estimates. Although [9] proposes the visual–inertial alignment method to estimate initial values (scale, velocity, gravity and gyroscope bias), the accelerometer bias is ignored and the initial values in the initial step are not accurate enough. The neglection of the accelerometer bias will decrease the accuracy of the estimated scale and gravity and further cause some serious problems in applications such as augmented reality, which require a high precision of tracking and mapping. The IMU initialization method proposed in [8] is able to estimate all the required initial parameters, but it lacks a termination criterion for IMU initialization, which results in an additional computational consumption. In addition, the gravity and accelerometer bias are estimated together, which may lead to inaccurate estimation, because the accelerometer bias is usually coupled with gravity and these are hard to distinguish under small rotation [20]. In summary, it is still a challenging problem to obtain a robust and accurate initialization method.

Therefore, in this paper we propose a more accurate initial state estimation approach to estimate visual scale, gravity, velocity and IMU parameters. The contributions of this paper are given in the following ways. Firstly, considering that the gravity magnitude is known, we propose a method to refine the estimated gravity by optimizing the 2D error state on its tangent space, then estimate the accelerometer bias separately. The accurate estimation of the accelerometer bias and gravity will improve the accuracy of the estimated scale and trajectory. Secondly, we put forward an automatic method to identify convergence and termination for visual–inertial initial state estimation, which will decrease the computational consumption of the initialization process.

The rest of this paper is organized as follows. In Section 2, we discuss the related visual–inertial systems and their corresponding initialization methods. We give a brief introduction about visual measurements, the IMU pre-integration technique, the camera model and the kinematics model of the IMU in Section 3. In Section 4, we describe our initialization approach that sequentially estimates the gyroscope bias, gravity vector, accelerometer bias, visual scale and velocity. Section 5 is dedicated to showing the performance of our approaches, and we compare the results with ones of the state-of-the-art approaches and the ground truth data. We conclude the paper in Section 6.

## 2. Related Work

Monocular visual–inertial SLAM systems have been a very active research topic in the field of robot navigation and augmented reality. A wealth of research work has been proposed [8,9,21,22,23]. The early visual–inertial SLAM algorithm [24] fuses visual and inertial measurements under a loosely coupled filter-based framework. After this, tightly coupled filter-based approaches [7,15] were applied for monocular visual–inertial SLAM. A drawback of using filter-based approaches is that it may lead to a suboptimal problem because of linearizing the estimated states early. With the progress of research and the improvement in computer performance, nonlinear optimization-based methods have been widely used in visual–inertial SLAM systems, which guarantee a higher accuracy. In [25], the authors describe a full smoothing method to estimate the entire history of the states by solving a large nonlinear optimization problem. While promising, it yields a high computational complexity, and its real-time performance gradually declines as the trajectory and the map grow over time. Most recently, the work presented in [10] applies a keyframe-based method to fuse visual–inertial measurements. Sliding window and marginalization techniques are utilized to ensure real-time operation and achieve remarkable success. Additionally, the IMU pre-integration technique proposed in [26] is able to form relative motion constrains by integrating inertial measurements between keyframes, which avoids computing the integration repeatedly whenever a linearization point changes. However, the performance of state-of-the-art nonlinear monocular visual–inertial systems heavily relies on the accuracy of the initial estimated states. A poor initial state estimation will decrease the convergence speed or even lead to completely incorrect estimates.

Therefore the initial state estimation is very important and attracts great interest among researchers. The early paper [27] presents a deterministic closed-form solution to compute the gravity and the visual scale and provide the initial metric values for the state estimation filter. However as a result of the lack of IMU biases, the estimated scale and gravity are not accurate, which results in a poor system stability. In [24], the scale, velocity and IMU biases are estimated as additional state variables under an EKF framework. However, the estimated variables are slow to converge to stable values. The authors of [28] put forward a loosely coupled visual–inertial system that assumes that MAVs need to take off nearly horizontally at the beginning so as to complete the initialization process. The initialization method proposed in [29] requires that the initial attitude should be aligned with the gravity direction. Without prior information, the above two approaches are not suitable for on-the-fly initialization. Moreover, the gyroscope bias is ignored in the initialization procedure of [17,20], which leads to inaccurate state estimation.

A pioneering work is proposed in [30]. The authors propose a lightweight visual–inertial initialization method. However, the IMU biases and scale need to be refined in the tracking thread. In visual–inertial ORB-SLAM2 [8], the authors propose a loosely coupled visual–inertial alignment method that can recover entire visual–inertial parameters. While promising, it lacks a robust termination criterion to automatically bootstrap the following SLAM algorithm. In addition, considering that the accelerometer bias is usually coupled with gravity under small rotation, estimating the gravity and accelerometer bias separately is a better solution.

For this reason, it is promising to propose a robust and complete initialization procedure that can obtain accurate initial values, particularly the visual scale and the gravity direction. Therefore this paper is dedicated to initializing the gravity and accelerometer bias separately. Additionally, we also present an automatic termination criterion for determining when the estimated values converge.

## 3. Visual–Inertial Preliminaries

**Figure 1:**
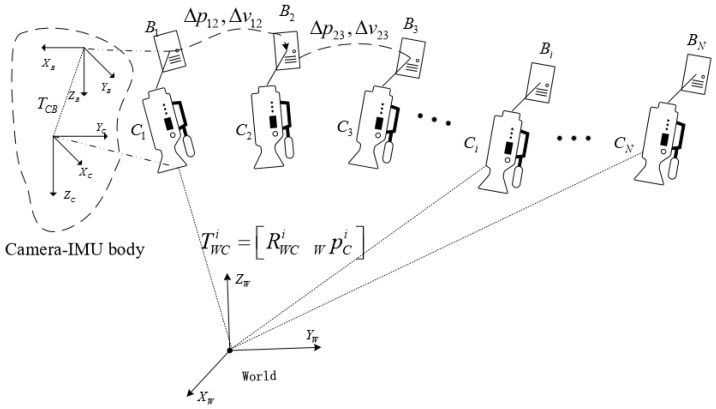
The relationship between different coordinate frames and multiple states of camera–IMU.

We consider a visual–inertial odometry problem [9] in which the state of a sensing system equipped with an IMU and a monocular camera need to be estimated in real-time. In this paper, we consider ${\left(\cdot \right)}_{C}$ as the camera frame, which is an arbitrary fixed frame in a visual structure. We define the first camera frame as the world frame ${\left(\cdot \right)}_{W}$. The IMU frame is aligned with the body frame ${\left(\cdot \right)}_{B}$, thus we regard the IMU frame as the body frame, which is irrelevant to the camera frame. The matrix $T_{CB}=\left[R_{CB}\;{}_{C}P_{B}\right]$ represents the transformation from the body frame B to the camera frame C, $R_{CB}$ is the rotational matrix and ${}_{C}P_{B}$ is the translation vector. We assume that the intrinsic parameters of the camera and extrinsic parameters between the camera and IMU are calibrated by using the methods of [31,32], respectively. In this section, we introduce some preliminary knowledge about visual measurements, the inertial sensor model, and IMU pre-integration. Figure 1 shows the situation of a camera–IMU setup with its corresponding coordinate frames. Multiple camera–IMU units represent the consecutive states at continuous time, which is convenient for understanding the equations illustrated in Section 4.2.

### 3.1. Visual Measurements

Visual–inertial odometry includes measurements from the camera and the IMU. Our visual measurement algorithm is based on visual ORB-SLAM2 [6], which includes three threads for tracking, local mapping and loop closing. For the process of the initial state estimation, we use the tracking thread and the local mapping thread. For each frame, the tracking thread is performed and decides whether the new frame can be considered as a keyframe. Once a new keyframe is generated, the corresponding IMU pre-integration can be computed iteratively by integrating all IMU measurements between two consecutive keyframes. At every frame, the camera can observe multiple landmarks. With the conventional pinhole-camera model [33], a 3D landmark $X_{c}\in R^{3}$ in the camera frame is mapped to the image coordinate $x\in R^{2}$ through a camera projection function $\pi :R^{3}\mapsto R^{2}$:

(1) $$\pi \left(X_{c}\right)=\left[\begin{array}{c} f_{u}\frac{x_{c}}{z_{c}}+c_{u}\\ f_{v}\frac{y_{c}}{z_{c}}+c_{v} \end{array}\right],X_{c}={\left[\begin{array}{ccc} x_{c} & y_{c} & z_{c} \end{array}\right]}^{T}$$

where ${\left[\begin{array}{cc} f_{u} & f_{v} \end{array}\right]}^{T}$ is the focal length and ${\left[\begin{array}{cc} c_{u} & c_{v} \end{array}\right]}^{T}$ is the principal point. Hence, by minimizing the re-projection error, we are able to recover the relative rotation and translation up to an unknown scale within multiple keyframes poses.

### 3.2. Inertial Measurements and Kinematics Model

An IMU generally integrates a 3-axis gyroscope sensor and a 3-axis accelerometer sensor, and correspondingly, the measurements provide us the angular velocity and the acceleration of the inertial sensor at a high frame rate with respect to the body frame B. The IMU measurement model contains two kinds of noise. One is white noise n(t); another is random walk noise that is a slowly varying sensor bias b(t). Thus we have

(2) $${}_{B}{\tilde{w}}_{WB}\left(t\right)={}_{B}w_{WB}\left(t\right)+b_{g}\left(t\right)+n_{g}\left(t\right)$$

(3) $${}_{B}\tilde{a}\left(t\right)=R_{WB}^{T}\left(t\right)\left({}_{W}a(t)-{}_{W}g\right)+b_{a}\left(t\right)+n_{a}\left(t\right)$$

where ${}_{B}\tilde{w}\left(t\right)$ and ${}_{B}\tilde{a}\left(t\right)$ are the measured angular velocity and acceleration values expressed in the body frame; the real angular velocity ${}_{B}w_{WB}\left(t\right)$ and the real acceleration ${}_{W}a(t)$ are what we need to estimate. $R_{WB}$ is the rotational part of the transformation matrix $\left[R_{WB}\;{}_{W}P_{B}\right]$, which maps a point from a body frame *B* to the world frame *W*. Generally, the dynamics of nonstatic bias $b_{g},b_{a}$ are modeled as a random process, which can be described as

(4) $${\dot{b}}_{g}=n_{b_{g}}\;{\dot{b}}_{a}=n_{b_{a}}$$

Here $n_{b_{g}}$ and $n_{b_{a}}$ are the zero-mean Gaussian white noise. We utilize the following IMU kinematics model commonly used in [34] to deduce the evolution of the pose and velocity of the body frame:

(5) $${}_{W}{\dot{R}}_{WB}=R_{WB}{}_{B}\omega^{\wedge }\;{}_{W}\dot{\nu }={}_{W}a\;{}_{W}\dot{p}={}_{W}\nu$$

where ${}_{W}{\dot{R}}_{WB}$, ${}_{W}\dot{\nu }$ and ${}_{W}\dot{p}$ respectively represent the derivatives of the rotation matrix $R_{WB}$, the velocity vector ${}_{W}\nu$ and the translation vector ${}_{W}p$ with respect to time. When we assume that ${}_{W}a$ and ${}_{B}\omega$ are constants in the time interval [t,t+Δt], the pose and velocity of the IMU at time [t,t+Δt] can be described as follows:

(6) $$R_{WB}\left(t+\Delta t\right)=R_{WB}\left(t\right)Exp\left({}_{B}\omega (t)\Delta t\right)$$

(7) $${}_{W}\nu (t+\Delta t)={}_{W}\nu (t)+{}_{W}a(t)\Delta t$$

(8) $${}_{W}p\left(t+\Delta t\right)={}_{W}p\left(t\right)+{}_{W}\nu \left(t\right)\Delta t+1/2{}_{W}a\left(t\right)\Delta t^{2}$$

Equations (6)–(8) can be further represented by using IMU measurements:

(9) $$R\left(t+\Delta t\right)=R\left(t\right)Exp(\left(\tilde{w}\left(t\right)-b_{g}\left(t\right)-n_{g}\left(t\right)\right)\Delta t)$$

(10) $$\nu \left(t+\Delta t\right)=\nu \left(t\right)+g\Delta t+R\left(t\right)(\tilde{a}\left(t\right)-b_{a}\left(t\right)-n_{a}\left(t\right))\Delta t$$

(11) $$p\left(t+\Delta t\right)=p\left(t\right)+\nu \left(t\right)\Delta t+1/2g\Delta t^{2}+1/2R\left(t\right)\left(\tilde{a}\left(t\right)-b_{a}\left(t\right)-n_{a}\left(t\right)\right)\Delta t^{2}$$

### 3.3. IMU Pre-Integration

From Equations (9)–(11), we can see that the IMU state propagation requires the rotation, position and velocity of the body frame. With the starting states changing, we need to re-propagate the IMU measurements, which is time consuming. To avoid this problem, we use the IMU pre-integration technique that is first proposed in [35] and is further extended to the manifold structure in [26]. Here we give a rough overview of its theory and usage within monocular visual–inertial SLAM systems. We assume that the IMU is synchronized with the camera and provides measurements at discrete times *k*. The relative motion increments between two consecutive keyframes at times k=i and k=j are defined as

(12) $$\Delta R_{ij}≐R_{i}^{T}R_{j}=\prod_{k=i}^{j-1}Exp\left(\left(\tilde{\omega_{k}}-{b_{g}}_{k}-{n_{g}}_{k}\right)\Delta t\right)$$

(13) $$\nu_{ij}≐R_{i}^{T}\left(\nu_{j}-\nu_{i}-g\Delta t_{ij}\right)=\sum_{k=i}^{j-1}\Delta R_{ik}\left(\tilde{a_{k}}-b_{ak}-n_{ak}\right)\Delta t$$

(14) $$\Delta p_{ij}≐R_{i}^{T}\left(p_{j}-p_{i}-\nu_{i}\Delta t_{ij}-1/2g\Delta t_{ij}^{2}\right)=\sum_{k=i}^{j-1}\left[\Delta \nu_{ik}\Delta t+1/2\Delta R_{ik}\left({\tilde{a}}_{k}-b_{ak}-n_{ak}\right)\Delta t^{2}\right]$$

In the above equations, the IMU biases are considered to be constants in the time interval Δt. However, more likely, the estimated biases change by a small amount δb during optimization. Therefore, the Jacobians $J_{\left(\cdot \right)}^{g}$ and $J_{\left(\cdot \right)}^{a}$ are applied to indicate how the measurements Δ(·) change with a change δb in the bias estimation; then the pose and velocity can be further expressed as

(15) $$R_{WB}^{i+1}=R_{WB}^{i}\Delta R_{i,i+1}Exp\left(J_{\Delta R}^{g}b_{g}^{i}\right)$$

(16) $${}_{W}\nu_{B}^{i+1}={}_{W}\nu_{B}^{i}+g{}_{W}\Delta t_{i,i+1}+R_{WB}^{i}\left(\Delta \nu_{i,i+1}+J_{\Delta \nu }^{g}b_{g}^{i}+J_{\Delta \nu }^{a}b_{a}^{i}\right)$$

(17) $${}_{W}p_{B}^{i+1}={}_{W}p_{B}^{i}+{}_{W}\nu_{B}^{i}\Delta t_{i,i+1}+0.5g_{W}\Delta t_{i,i+1}^{2}+R_{WB}^{i}\left(\Delta p_{i,i+1}+J_{\Delta p}^{a}b_{a}^{i}\right)$$

Here the IMU pre-integration is computed iteratively when IMU measurements arrive, and the Jacobians can be precomputed during the pre-integration with the method mentioned in [26].

## 4. Visual–Inertial Initial State Estimation

**Figure 2:**
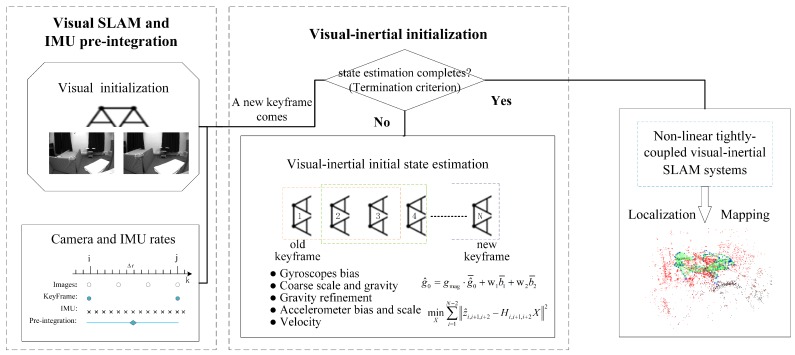
Our visual–inertial initial state estimation algorithm.

In this section, we detail the complete process of our initial state estimation algorithm, which sequentially estimates the gyroscope bias, gravity vector (including gravity refinement), accelerometer bias, metric scale and velocity. An overview of our method is given in Figure 2. Our algorithm first only uses visual measurements as in the ORB-SLAM2 [6] for a few keyframes. The corresponding IMU pre-integration between these keyframes are computed at the same time. These two steps have been detailed in Section 3. When a new keyframe is created, we run our loosely coupled visual–inertial initial state estimation algorithm to iteratively update the gyroscope bias, gravity vector, accelerometer bias, metric scale and velocity sequentially. This procedure continues until the termination criterion is achieved.

In our loosely coupled visual–inertial initial state estimation module, we first recover the gyroscope bias and then roughly estimate the gravity vector and scale without considering the accelerometer bias. Because the gravity norm is usually known (∼9.8 m/s${}^{2}$), we refine the estimated gravity vector by optimizing the 2D error state on its tangent space. After the gravity refinement, we regard it as a fixed vector. Then we begin to accurately estimate the scale and accelerometer bias. Finally we compute the velocities of all keyframes. This is the same as the IMU initialization process of [8] in the first two steps. The main differences are reflected in the remaining steps. In our method, we are dedicated to estimating the gravity and accelerometer bias separately, these are normally difficult to distinguish from each other under the small rotation condition. Furthermore, we constrain the magnitude to refine the estimated gravity vector. In addition, because the condition number can indicate whether a problem is well conditioned, we regard it as one of the termination indicators. Once the termination criterion is achieved, the initialization process will be automatically terminated. The estimated initial state values can be used to launch the nonlinear tightly coupled visual–inertial SLAM system. To sum up, our initial state estimation procedure is partly based on the IMU initialization of [8], but we further improve the method and provide a more accurate and complete initialization procedure.

### 4.1. Gyroscope Bias Estimation

Considering two consecutive keyframes *i* and i+1 in the keyframe database, we have their orientations $R_{WC}^{i}$ and $R_{WC}^{i+1}$ from visual ORB-SLAM2, as well as their integration $\Delta R_{i,i+1}$ from the IMU pre-integration. We estimate the gyroscope bias $b_{g}$ by minimizing the residual errors between the relative rotation from the vision and gyroscope integration. The detailed derivation of Equation (18) can be found in [26].

(18) $$\underset{b_{g}}{\arg\min}\sum_{i=1}^{N-1}||Log\left({\left(\Delta R_{i,i+1}Exp\left(J_{\Delta R}^{g}b_{g}\right)\right)}^{T}R_{BW}^{i+1}R_{WB}^{i}\right){\left|\right|}^{2}$$

In Equation (18), *N* is the number of keyframes and $J_{\Delta R}^{g}$ denotes the first-order approximation of the impact of the changing gyroscope bias. $R_{WB}^{\left(\cdot \right)}=R_{WC}^{\left(\cdot \right)}R_{CB}$, which can be computed by transforming the pose of the IMU to the world coordinate system. By solving Equation (18) by the Gauss–Newton method, we can obtain the estimated gyroscope bias $b_{g}$. Because the initial gyroscope bias is set to zero at the beginning, we now update the pre-integration $\Delta R_{ij}$, $\Delta \nu_{ij}$ and $\Delta p_{ij}$ with respect to the estimated $b_{g}$.

### 4.2. Coarse Scale and Gravity Estimation

With small rotation, the accelerometer bias is difficult to be distinguished from gravity. Therefore the second step of our initialization process is to coarsely estimate the preliminary scale *s* and gravity $g_{0}$ without regard to the accelerometer bias $b_{a}$. We define the variables that we want to estimate as

(19) $$X_{s,g_{0}}={\left[s,g_{0}\right]}^{T}\in R^{4x1}$$

Because of the scale ambiguity existing in monocular visual SLAM systems, an additional visual scale *s* is necessary when transforming the position in the camera frame *C* to the body frame *B*, which is expressed as

(20) $${}_{W}p_{B}=s_{W}p_{C}+R_{WC}{}_{C}p_{B}$$

We substitute Equation (20) into Equation (17), which represents the relative position relation between two consecutive keyframes *i* and i+1. Without considering the effect of the accelerometer bias, we can obtain

(21) $$\begin{array}{c} \left[\Delta p_{i,i+1}-{R_{WB}^{i}}^{T}{\left(R_{WC}^{i+1}-R_{WC}^{i}\right)}_{c}p_{B}\right]=\left[\begin{array}{ccc} -{R_{WB}^{i}}^{T}\Delta t_{i,i+1} & {R_{WB}^{i}}^{T}\left({}_{W}p_{C}^{i+1}-{}_{W}p_{C}^{i}\right) & -\;0.5{R_{WB}^{i}}^{T}\Delta t_{i,i+1} \end{array}\right]\left[\begin{array}{c} \nu_{i}\\ s\\ g_{0} \end{array}\right] \end{array}$$

If stacking all equations between every two consecutive keyframes using Equation (21), there will be N−1 velocities that need to be solved. This would lead to a high computational complexity. Therefore in this section we do not solve the velocities of *N* keyframes. On the contrary, we consider Equation (21) between three consecutive keyframes (Figure 1 shows an example) and exploit the velocity Equation (13):

(22) $$\begin{array}{cc} {\hat{z}}_{i,i+1,i+2} & =[{\left(R_{WC}^{i}-R_{WC}^{i+1}\right)}_{C}p_{B}\Delta t_{i+1,i+2}-{\left(R_{WC}^{i+1}-R_{WC}^{i+2}\right)}_{C}p_{B}\Delta t_{i,i+1}\\ & -R_{WB}^{i+1}\Delta p_{i+1,i+2}\Delta t_{i,i+1}-R_{WB}^{i}\Delta \nu_{i,i+1}\Delta t_{i,i+1}\Delta t_{i+1,i+2}+R_{WB}^{i}\Delta p_{i,i+1}\Delta t_{i+1,i+2}]\\ & =\left[\begin{array}{cc} \left({}_{W}p_{C}^{i+1}-{}_{W}p_{C}^{i}\right)\Delta t_{i+1,i+2}-\left({}_{W}p_{C}^{i+2}-{}_{W}p_{C}^{i+1}\right)\Delta t_{i,i+1} & 0.5I_{3x3}\left(\Delta t_{i,i+1}^{2}\Delta t_{i+1,i+2}+\Delta t_{i+1,i+2}^{2}\Delta t_{i,i+1}\right) \end{array}\right]\left[\begin{array}{c} s\\ g_{0} \end{array}\right]\\ & =H_{i,i+1,i+2}X_{s,g_{0}} \end{array}$$

In the above formula, ${}_{W}p_{c}^{\left(\cdot \right)}$ and $R_{WC}^{\left(\cdot \right)}$ are obtained from ORB-SLAM2, $\Delta p_{\left(\cdot \right)}$ and $\Delta \nu_{\left(\cdot \right)}$ are from the IMU pre-integration, and $\Delta t_{i,i+1}$ is the time interval between two consecutive keyframes. Stacking every three consecutive keyframes using Equation (22), we can form the following least-square problem. Solving this, we can obtain the coarsely estimated gravity vector ${\hat{g}}_{0}$ and scale *s*.

(23) $$\underset{X_{s,g_{0}}}{min}\sum_{i=1}^{N-2}\left|\right|{\hat{z}}_{i,i+1,i+2}-H_{i,i+1,i+2}X_{s,g_{0}}{\left|\right|}^{2}$$

### 4.3. Gravity Refinement

Because the gravity norm is known in most cases, the gravity vector only has 2 degrees of freedom. On the basis of this, the estimated gravity ${\hat{g}}_{0}$ obtained from Section 4.2 can be further refined. If the additional gravity norm constraint is straightway added into the optimization problem in Equation (23), it will become a nonlinear system that is hard to solve. Therefore, we enforce the gravity magnitude by optimizing the 2D error state on its tangent space, similarly to [30].

**Figure 3:**
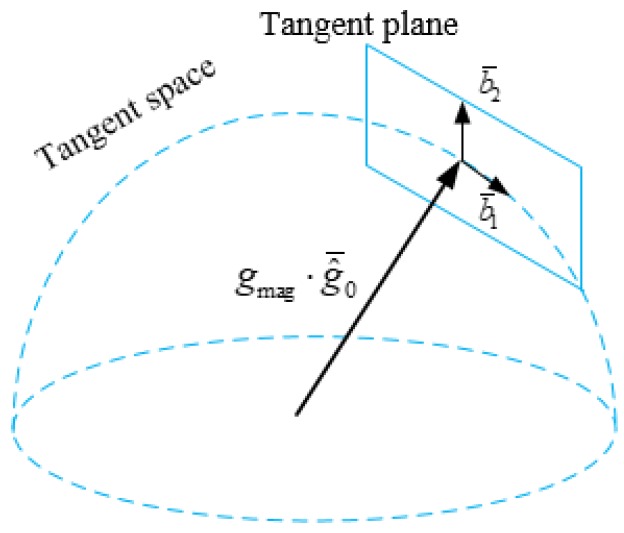
The tangent space model of gravity. The gravity magnitude is the radius of a sphere.

As shown in Figure 3, the estimated gravity can be re-parameterized as

(24) $${\hat{g}}_{0}=g_{mag}\cdot \bar{\hat{g_{0}}}+w_{1}{\bar{b}}_{1}+w_{2}{\bar{b}}_{2}$$

where $g_{mag}$ is the known gravity magnitude, $\bar{\hat{g_{0}}}$ is the direction of the current estimated gravity ${\hat{g}}_{0}$, and ${\bar{b}}_{1}$ and ${\bar{b}}_{2}$ are two orthogonal bases on the tangent plane; $w_{1}$ and $w_{2}$ are the corresponding 2D components that need to be estimated. It is easy to find one set of ${\bar{b}}_{1}$ and ${\bar{b}}_{2}$ using the Gram–Schmidt process. Then we replace gravity ${\hat{g}}_{0}$ in Equation (22) with Equation (24). In this way, we can form a least-square problem similar to Equation (23) and solve it via Singular Value Decomposition (SVD). Then we iterate these steps several times until the estimated ${\hat{g}}_{0}$ converges.

### 4.4. Accelerometer Bias and Scale Estimation

After refining the gravity vector, we regard it as a fixed vector $g_{W}$ in the world frame. In Section 4.2, we do not consider the accelerometer bias. The estimated scale *s* may be coarse, and thus we estimate the accelerometer bias $b_{a}$ and scale *s* together in this step using Equation (17). The variables that we would like to estimate are defined as

(25) $$X_{s,b_{a}}={\left[s,b_{a}\right]}^{T}\in R^{4x1}$$

Now adding the accelerometer bias into Equation (21), it becomes

(26) $$\begin{array}{c} \left[\Delta p_{i,i+1}-{R_{WB}^{i}}^{T}{\left(R_{WC}^{i+1}-R_{WC}^{i}\right)}_{C}p_{B}+0.5{R_{WB}^{i}}^{T}g_{W}\Delta t_{i,i+1}^{2}\right]=\left[\begin{array}{ccc} -{R_{WB}^{i}}^{T}\Delta t_{i,i+1} & {R_{WB}^{i}}^{T}\left({}_{W}p_{C}^{i+1}-{}_{W}p_{C}^{i}\right) & -\;J_{\Delta p}^{a} \end{array}\right]\left[\begin{array}{c} \nu_{i}\\ s\\ b_{a} \end{array}\right] \end{array}$$

The Jacobian $J_{\left(\cdot \right)}^{a}$ denotes a first-order approximation of the impact of the changing accelerometer bias. Similarly to the method described in Section 4.2, we can obtain the estimated accelerometer bias $b_{a}$ and scale *s*.

### 4.5. Velocity Estimation

So far, we have estimated all variables except the velocity. In other words, the velocity is the only unknown in Equation (26). Therefore we can compute the velocities of the first N−1 keyframes using Equation (26), then compute the velocity of the last keyframe using Equation (13).

### 4.6. Termination Criterion

In our method the visual–inertial initialization process is automatically terminated when all estimated states are convergent. Because the norm of the nominal gravity is a constant 9.806 m/s${}^{2}$, we regard it as one of the convergence indicators. Another we use here is the condition number, which can indicate whether the problem is well conditioned. Once the visual–inertial initialization is successful, all 3D points in the map and the position of keyframes are updated according to the estimated scale. Because the IMU parameters have been estimated, we can integrate all IMU measurements to predict the next camera pose.

## 5. Experimental Results

In order to evaluate the performance of our initial state estimation approach, the public EuRoC dataset [36] was used. The EuRoC dataset consists of 11 sequences of 2 scenes in the Vicon room and industrial machine hall, and it provides synchronized global shutter stereo images at 20 Hz with IMU measurements at 200 Hz and trajectory ground truth. We only used one camera image set and IMU measurements to conduct the experiments in a virtual machine with 2 GB of RAM.

Because the EuRoC dataset does not provide an explicit ground truth scale, we need to calculate the true scale according to the ground truth data and the trajectory generated from visual ORB-SLAM2. Once the initialization of ORB-SLAM2 system completes, it produces an initial translation between the first two keyframes. After this, we can calculate the true translation on the basis of their corresponding ground truth states. Then the true scale (benchmark scale) will be the ratio of the true translation to the initial translation.

### 5.1. The Performance of Visual–Inertial Initial State Estimation

**Figure 4:**
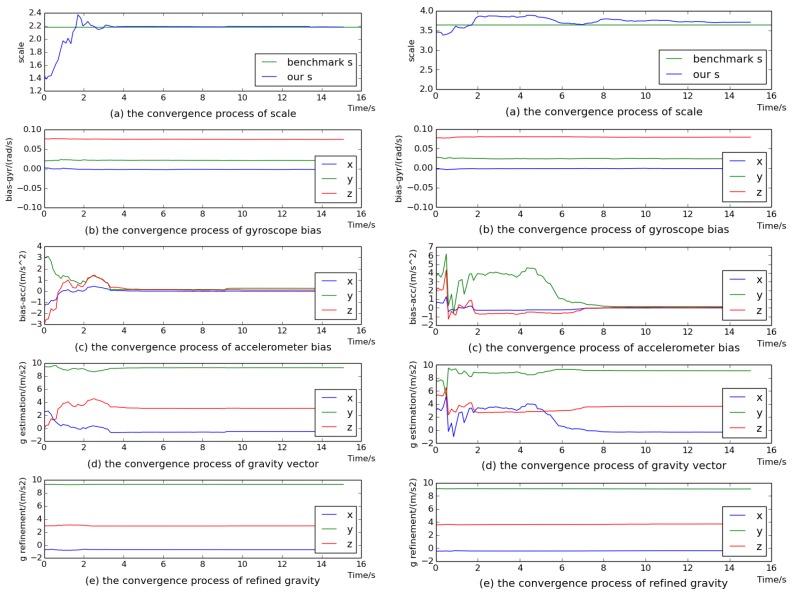
The convergence procedure of initial state on sequences V1_02_medium (**left**) and V2_02_medium (**right**).

**Figure 5:**
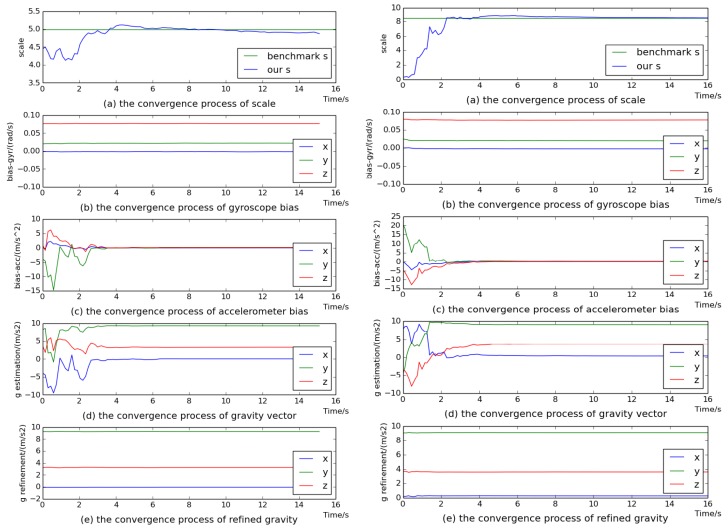
The convergence procedure of initial state on sequences MH_03_medium (**left**) and MH_04_difficult (**right**).

Here, we use the sequences of two scenes for evaluation. The variables of gyroscope bias, gravity vector, visual scale and accelerometer bias are sequentially estimated. Figure 4 and Figure 5 show the convergence process of all the estimated variables on sequences V1_02_medium, V2_02_medium, MH_03_medium and MH_04_difficult. We can see that all variables converged to stable values after 8 s. Even on sequence V1_02_medium, all variables converged quickly after 5 s. In particular, the estimated visual scale was quite close to the benchmark scale. From Figure 4b,c and Figure 5b,c, it can be seen that the gyroscope bias converged quickly and the accelerometer bias converged to almost 0. Figure 4d and Figure 5d demonstrate the convergence process of the estimated gravity vector, whose three components seriously deviated from stable values within 6 s, while Figure 4e and Figure 5e show that the components of the refined gravity vector quickly converged to final steady-state values only after 2 s. Thus it can be indicated that our gravity refinement approach can efficiently speed up the convergence process of the estimated gravity vector.

### 5.2. The Accuracy of Scale Estimation

In this section, we evaluate the accuracy of the estimated scale using our method in two scenes of the EuRoC dataset including sequences V1_01_easy, V2_01_easy, V1_02_medium, V2_02_medium, MH_01_easy, MH_02_easy, MH_03_medium and MH_04_difficult. In order to effectively verify the accuracy and reliability of our approach, the visual measurements started without any prior information. Table 1 indicates the testing results on eight sequences. Compared with the state-of-the-art visual–inertial ORB-SLAM2 [8], the estimated scale using our method outperformed it on seven test sequences. The scale estimation error of our method was less than 5% on five sequences, and some of them were quite close to the benchmark scale. The scale error was under 7% on the sequences V2_02_medium, MH_02_easy and MH_04_difficult with a bright scene; in particular, the scales estimated by our approach achieved a higher precision than those from [8] in the mass. On sequence V2_01_easy, the results of [8] were better than ours, but fortunately, our approach also achieved a high accuracy, with an error below 5%.

### 5.3. The Effect of Termination Criterion

**Figure 6:**
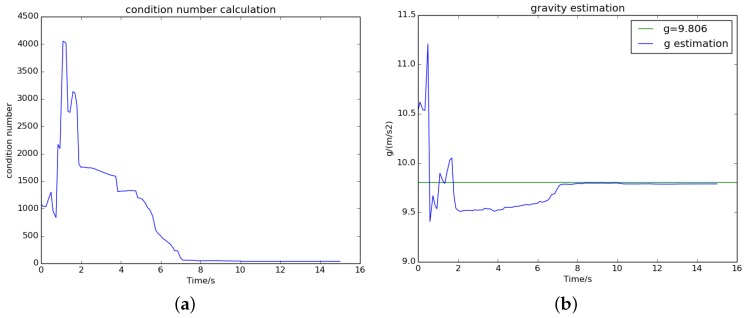
The convergence process of (**a**) the condition number and (**b**) the estimated gravity on sequence V2_02_medium.

**Figure 7:**
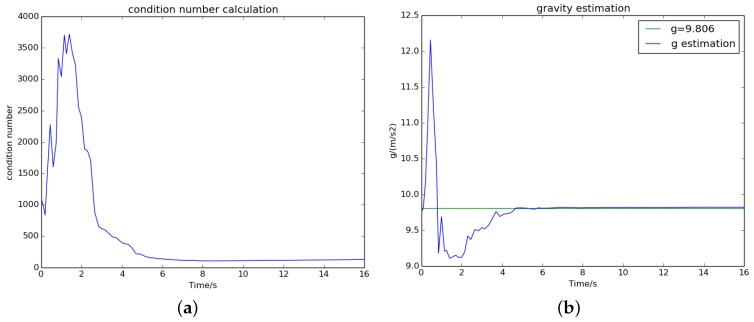
The convergence process of (**a**) the condition number and (**b**) the estimated gravity on sequence MH_04_difficult.

The visual–inertial initialization process continues until both the termination criteria are achieved. For the sequences V2_02_medium and MH_04_difficult, Figure 6 and Figure 7 show that the condition number dropped to a small and stable value after 8 and 6 s, respectively, which means that we obtain a well-conditioned problem. Meanwhile, the norm of the estimated gravity (blue) converged to almost the nominal gravity (green). On the right side of Figure 4 and Figure 5, we can see that all estimated variables were convergent after 8 and 6 s. This proves that the termination criteria are valid.

### 5.4. The Tracking Accuracy of Keyframes

**Figure 8:**
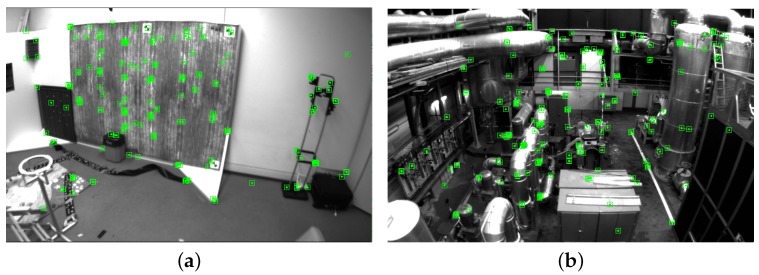
The representative images of two scenes: (**a**) the Vicon room and (**b**) the machine hall.

**Figure 9:**
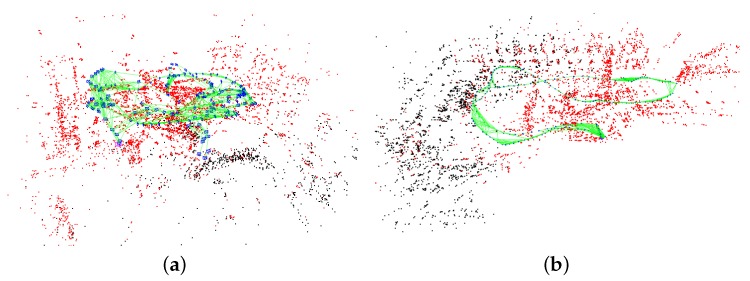
The reconstructed sparse map of (**a**) the Vicon room and (**b**) the machine hall.

Once we have estimated a stable and accurate scale, the initialization procedure terminates. All 3D points in the map and the positions of keyframes are updated according to the estimated scale. The estimated IMU parameters can be used to launch the nonlinear tightly coupled visual–inertial SLAM system. Figure 8 shows the processed images of the Vicon room and the industrial machine hall. The final reconstructed sparse map corresponding to the above two scenes is presented in Figure 9.

**Figure 10:**
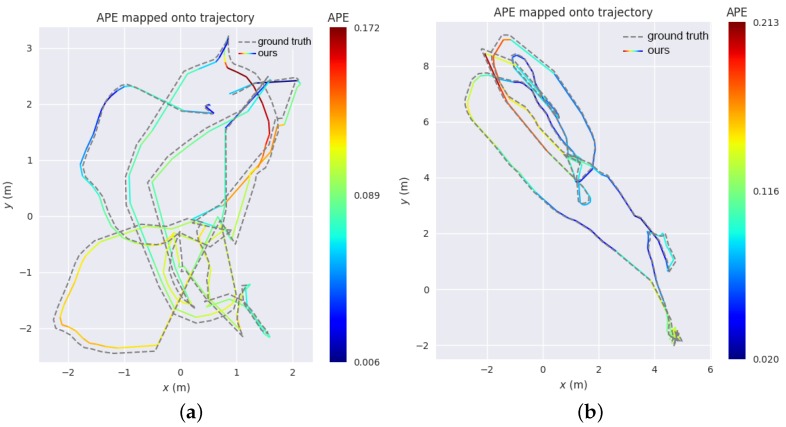
The trajectory of keyframes on sequences (**a**) V1_01_easy and (**b**) MH_01_easy of two scenes. The colorful trajectory is produced by combining our initial state estimation method with visual–inertial ORB-SLAM2 back-end; the ground truth trajectory is provided by EuRoC dataset. The various colors express the range of the corresponding absolute pose error (APE).

Because the evo (https://michaelgrupp.github.io/evo/) package provides a small library for handling and evaluating the trajectory of odometry and SLAM algorithms, we made use of this open-source tool to evaluate the trajectory accuracy of visual–inertial SLAM initialized with our algorithm. Figure 10 illustrates the trajectory of keyframes computed by combining our initialization method with the visual–inertial ORB-SLAM2 back-end, which is close to the ground truth trajectory provided by the EuRoC dataset. The colormap reveals the relationship between the colors and the absolute pose error (APE). As shown in Figure 10a, the corresponding pose error for sequence V1_01_easy varied from the minimum, 0.0062 m, to the maximum, 0.1715 m. The values of the mean error (ME), root mean square error (RMSE) and sum of squares error (SSE) were 0.0913 m, 0.0972 m and 1.4839 m${}^{2}$ respectively. Figure 10b also shows the APE tested on sequence MH_01_easy; the corresponding ME, RMSE and SSE were 0.094816 m, 0.102795 m, and 2.018254 m${}^{2}$. Thus it can be concluded that visual–inertial SLAM initialized with our initial state estimation algorithm is able to recover the metric scale and does not suffer from scale drift.

We compared the tracking performance of our method with those of state-of-the-art methods [8] and [6] on EuRoC dataset. The above systems could process all sequences, except V1_03_difficult and V2_03_difficult, for which the movement was so intense that the system could not survive. On each sequence, we tested five times and used the evo package to calculate the relative pose error (RPE) by aligning the estimated trajectory with the ground truth; we show the average results of the translation ME, RMSE and SSE in Table 2. From Table 2, we can see that the results of our approach were worse than those of [6] for six sequences. This was because the tightly coupled nonlinear optimization for visual–inertial fusion is more complex and costs more time, as there are nine additional states (IMU biases and velocity) for each keyframe. In order to achieve real-time performance, the local window size for local bundle adjustment of visual–inertial ORB-SLAM2 initialized with our method has to be smaller than that of [6], which would result in a decrease of the optimized states of keyframes and map points and further cause reduced accuracy of the trajectory and map. However, comparing the results from [8] with ours, we can clearly see that our initial state estimation approach could improve the tracking accuracy for six sequences, which were V1_01_easy, V2_02_medium, MH_01_easy, MH_02_easy, MH_04_difficult, and MH_05_difficult.

## 6. Conclusions

In this paper, we propose a more accurate algorithm for initial state estimation in a monocular visual–inertial SLAM system. The main contributions of our initialization method are given in the following ways. Firstly, considering that the gravity magnitude is known, we propose a method to refine the estimated gravity by optimizing the 2D error state on its tangent space. Then we estimate the accelerometer bias with the refined gravity fixed. Secondly, we propose an automatic way to determine when to terminate the process of visual–inertial initialization. On the whole, we present a complete and robust initialization method and provide accurate initial values (scale, gravity vector, velocity and IMU biases) to bootstrap the nonlinear visual–inertial SLAM framework. We verify the effectiveness of the algorithm on all sequences in two scenes of the public EuRoC dataset. Experimental results show that the proposed methods can achieve accurate initial state estimation, the gravity refinement approach can efficiently speed up the convergence process of the estimated gravity vector, and the termination criterion performs well.

## Figures and Tables

**Table 1:** The results of scale estimation, compared with the scale from visual–inertial ORB-SLAM2 (VI ORB-SLAM2) [8] and benchmark scale after VI ORB-SLAM2 system runs for 15 s. The fifth column shows the percentage of error between the estimated scale using our method and the benchmark scale. The numbers in bold represent the estimated scale is more close to the benchmark scale.

**V1_01_Easy**					**V1_02_Medium**				
**No.**	**VI ORB-SLAM2**	**Ours**	**Benchmark Scale**	**Error**	**No.**	**VI ORB-SLAM2**	**Ours**	**Benchmark Scale**	**Error**
1	2.19802	**2.22213**	2.31443	3.98%	1	**2.28028**	2.11539	2.22096	4.75%
2	2.18622	**2.21418**	2.28095	2.93%	2	2.21166	**2.17452**	2.18273	0.38%
3	2.12814	**2.14899**	2.19818	2.24%	3	2.32011	**2.29834**	2.24939	2.18%
4	2.32220	**2.32414**	2.43320	4.48%	4	2.46152	**2.41389**	2.43513	0.87%
5	**2.11896**	2.14095	2.04617	4.63%	5	2.29164	**2.24925**	2.24915	0.00%
**V2_01_easy**					**V2_02_medium**				
**No.**	**VI ORB-SLAM2**	**Ours**	**Benchmark Scale**	**Error**	**No.**	**VI ORB-SLAM2**	**Ours**	**Benchmark Scale**	**Error**
1	3.15119	**3.13984**	3.09290	1.52%	1	3.72664	**3.66760**	3.47209	5.63%
2	**3.15596**	3.18330	3.04272	4.62%	2	3.71125	**3.64681**	3.59466	1.45%
3	**2.97907**	2.92119	2.96395	1.44%	3	3.57335	**3.53126**	3.47022	1.76%
4	**3.11335**	3.11445	3.06949	1.46%	4	3.52077	**3.41453**	3.21689	6.14%
5	**2.91192**	2.90283	2.95193	1.66%	5	3.78522	**3.67040**	3.44327	6.60%
**MH_01_easy**					**MH_02_easy**				
**No.**	**VI ORB-SLAM2**	**Ours**	**Benchmark Scale**	**Error**	**No.**	**VI ORB-SLAM2**	**Ours**	**Benchmark Scale**	**Error**
1	1.38302	**1.36595**	1.35822	0.57%	1	3.9205	**3.97242**	4.23094	6.11%
2	3.54077	**3.51395**	3.50519	0.25%	2	**4.09284**	4.05315	4.30175	5.78%
3	**3.28325**	3.25925	3.39144	3.90%	3	**3.26533**	3.25786	3.49253	6.72%
4	**4.30154**	4.27641	4.43791	3.64%	4	1.37276	**1.39001**	1.47774	5.94%
5	3.87869	**3.88449**	4.03829	3.81%	5	3.32629	**3.35212**	3.57335	6.19%
**MH_03_medium**					**MH_04_difficult**				
**No.**	**VI ORB-SLAM2**	**Ours**	**Benchmark Scale**	**Error**	**No.**	**VI ORB-SLAM2**	**Ours**	**Benchmark Scale**	**Error**
1	3.51556	**3.53472**	3.67447	3.80%	1	2.15634	**2.16695**	2.20023	1.51%
2	4.12347	**4.21518**	4.35231	3.15%	2	1.88379	**1.92157**	2.05139	6.32%
3	4.87332	**4.96042**	4.983	0.45%	3	1.14818	**1.19114**	1.22704	2.93%
4	**5.35339**	5.34029	5.43041	1.66%	4	8.52259	**8.51992**	8.47516	0.53%
5	5.17706	**5.18087**	5.35175	3.19%	5	**2.2521**	2.26677	2.13573	6.13%

**Table 2:** The accuracy of keyframe trajectories generated by visual–inertial ORB-SLAM2 (VI ORB- SLAM2) [8], ORB-SLAM2 [6] and VI ORB-SLAM2 system initialized with our initialization approach on EuRoC dataset with 11 sequences. The corresponding values of the mean error (ME), root mean square error (RMSE) and sum of squares error (SSE) are listed as follows.

Sequence	Ours			VI ORB-SLAM2			ORB-SLAM2		
	ME (m)	RMSE (m)	SSE (m^2^)	ME (m)	RMSE (m)	SSE (m^2^)	ME (m)	RMSE (m)	SSE (m^2^)
V1_01_easy	0.3522	0.5214	43.0723	0.3574	0.5293	44.4517	0.3119	0.4549	31.5616
V1_02_medium	0.4407	0.6515	53.7404	0.4321	0.6069	58.1439	0.4022	0.5256	43.0167
V1_03_difficult	×	×	×	×	×	×	×	×	×
V2_01_easy	0.1868	0.2315	8.7623	0.1876	0.2293	8.5764	0.1711	0.2208	8.1043
V2_02_medium	0.3361	0.6166	39.4145	0.3538	0.6151	40.3685	0.4316	0.6523	94.1142
V2_03_difficult	×	×	×	×	×	×	×	×	×
MH_01_easy	0.3727	0.5512	59.1512	0.3773	0.5605	61.0061	0.3399	0.4861	47.2790
MH_02_easy	0.2876	0.4018	30.7535	0.3276	0.4589	38.1945	0.3301	0.4727	40.7942
MH_03_medium	0.6190	0.9968	216.467	0.5960	1.0918	175.914	0.6939	1.0975	193.548
MH_04_difficult	0.5646	0.7044	89.1064	0.5745	0.8837	123.787	0.4581	0.5573	58.0247
MH_05_difficult	0.5477	0.6724	86.5826	0.5730	0.7036	90.0694	0.4589	0.5716	63.9264

## References

[1] Klein G., Murray D. (2007). Parallel tracking and mapping for small AR workspaces. Proceedings of the 2007 6th IEEE and ACM International Symposium on Mixed and Augmented Reality.

[2] Eade E., Drummond T. Unified Loop Closing and Recovery for Real Time Monocular SLAM. Proceedings of the 2008 19th British Machine Vision Conference, BMVC 2008.

[3] Davison A.J. Real-time simultaneous localisation and mapping with a single camera. Proceedings of the 2003 Ninth IEEE International Conference on Computer Vision.

[4] Davison A.J., Reid I.D., Molton N.D., Stasse O. (2007). MonoSLAM: Real-time single camera SLAM. IEEE Trans. Pattern Anal. Mach. Intell..

[5] Forster C., Pizzoli M., Scaramuzza D. (2014). SVO: Fast semi-direct monocular visual odometry. Proceedings of the 2014 IEEE International Conference on Robotics and Automation (ICRA).

[6] Mur-Artal R., Tardós J.D. (2017). Orb-slam2: An open-source slam system for monocular, stereo, and rgb-d cameras. IEEE Trans. Robot..

[7] Mourikis A.I., Roumeliotis S.I. A Multi-State Constraint Kalman Filter for Vision-aided Inertial Navigation. Proceedings of the IEEE International Conference on Robotics and Automation.

[8] Mur-Artal R., Tardós J.D. (2017). Visual-Inertial Monocular SLAM With Map Reuse. IEEE Robot. Autom. Lett..

[9] Li P., Qin T., Hu B., Zhu F., Shen S. (2017). Monocular visual-inertial state estimation for mobile augmented reality. Proceedings of the 2017 IEEE International Symposium on Mixed and Augmented Reality (ISMAR).

[10] Leutenegger S., Lynen S., Bosse M., Siegwart R., Furgale P. (2015). Keyframe-based visual-inertial odometry using nonlinear optimization. Int. J. Robot. Res..

[11] Usenko V., Engel J., Stückler J., Cremers D. Direct visual-inertial odometry with stereo cameras. Proceedings of the IEEE International Conference on Robotics and Automation.

[12] Concha A., Loianno G., Kumar V., Civera J. Visual-inertial direct SLAM. Proceedings of the IEEE International Conference on Robotics and Automation.

[13] Jones E.S., Soatto S. (2011). Visual-inertial navigation, mapping and localization: A scalable real-time causal approach. Int. J. Robot. Res..

[14] Wu K., Ahmed A., Georgiou G., Roumeliotis S. A Square Root Inverse Filter for Efficient Vision-aided Inertial Navigation on Mobile Devices. Proceedings of the Robotics: Science and Systems.

[15] Bloesch M., Omari S., Hutter M., Siegwart R. (2015). Robust visual inertial odometry using a direct EKF-based approach. Proceedings of the 2015 IEEE/RSJ International Conference on Intelligent Robots and Systems (IROS).

[16] Strasdat H., Montiel J., Davison A.J. (2010). Real-time monocular SLAM: Why filter?. Proceedings of the 2010 IEEE International Conference on Robotics and Automation (ICRA).

[17] Yang Z., Shen S. (2017). Monocular Visual–Inertial State Estimation With Online Initialization and Camera–IMU Extrinsic Calibration. IEEE Trans. Autom. Sci. Eng..

[18] Shen S., Michael N., Kumar V. Tightly-coupled monocular visual-inertial fusion for autonomous flight of rotorcraft MAVs. Proceedings of the IEEE International Conference on Robotics and Automation.

[19] Hesch J.A., Kottas D.G., Bowman S.L., Roumeliotis S.I. (2014). Consistency Analysis and Improvement of Vision-aided Inertial Navigation. IEEE Trans. Robot..

[20] Martinelli A. (2014). Closed-form solution of visual-inertial structure from motion. Int. J. Comput. Vis..

[21] Engel J., Sturm J., Cremers D. (2014). Scale-aware navigation of a low-cost quadrocopter with a monocular camera. Robot. Auton. Syst..

[22] Weiss S., Achtelik M.W., Lynen S., Chli M. Real-time onboard visual-inertial state estimation and self-calibration of MAVs in unknown environments. Proceedings of the IEEE International Conference on Robotics and Automation.

[23] Tanskanen P., Kolev K., Meier L., Camposeco F., Saurer O., Pollefeys M. Live Metric 3D Reconstruction on Mobile Phones. Proceedings of the IEEE International Conference on Computer Vision.

[24] Weiss S., Siegwart R. Real-time metric state estimation for modular vision-inertial systems. Proceedings of the IEEE International Conference on Robotics and Automation.

[25] Bryson M., Johnson-Roberson M., Sukkarieh S. Airborne smoothing and mapping using vision and inertial sensors. Proceedings of the IEEE International Conference on Robotics and Automation.

[26] Forster C., Carlone L., Dellaert F., Scaramuzza D. (2015). On-Manifold Preintegration for Real-Time Visual–Inertial Odometry. IEEE Trans. Robot..

[27] Kneip L., Weiss S., Siegwart R. Deterministic initialization of metric state estimation filters for loosely-coupled monocular vision-inertial systems. Proceedings of the IEEE/RSJ International Conference on Intelligent Robots and Systems.

[28] Faessler M., Fontana F., Forster C., Scaramuzza D. Automatic re-initialization and failure recovery for aggressive flight with a monocular vision-based quadrotor. Proceedings of the IEEE International Conference on Robotics and Automation.

[29] Weiss S., Brockers R., Albrektsen S., Matthies L. Inertial Optical Flow for Throw-and-Go Micro Air Vehicles. Proceedings of the IEEE Winter Conference on Applications of Computer Vision.

[30] Qin T., Shen S. Robust Initialization of Monocular Visual-Inertial Estimation on Aerial Robots. Proceedings of the 2017 IEEE/RSJ International Conference on Intelligent Robots and Systems (IROS).

[31] Zhang Z. (2000). A Flexible New Technique for Camera Calibration. IEEE Trans. Pattern Anal. Mach. Intell..

[32] Furgale P., Rehder J., Siegwart R. Unified temporal and spatial calibration for multi-sensor systems. Proceedings of the IEEE/RSJ International Conference on Intelligent Robots and Systems.

[33] Hartley R., Zisserman A. (2003). Multiple View Geometry in Computer Vision.

[34] Farrell J. (2008). Aided Navigation: GPS with High Rate Sensors.

[35] Lupton T., Sukkarieh S. (2012). Visual-Inertial-Aided Navigation for High-Dynamic Motion in Built Environments Without Initial Conditions. IEEE Trans. Robot..

[36] Burri M., Nikolic J., Gohl P., Schneider T., Rehder J., Omari S., Achtelik M.W., Siegwart R. (2016). The EuRoC micro aerial vehicle datasets. Int. J. Robot. Res..

